# The effect of dexmedetomidine on neuroprotection in pediatric cardiac surgery patients: study protocol for a prospective randomized controlled trial

**DOI:** 10.1186/s13063-022-06217-9

**Published:** 2022-04-08

**Authors:** Sang-Hwan Ji, Pyoyoon Kang, In-Sun Song, Young-Eun Jang, Ji-Hyun Lee, Jin-Tae Kim, Hee-Soo Kim, Eun-Hee Kim

**Affiliations:** Department of Anesthesiology and Pain Medicine, Seoul National University Hospital, Seoul National University College of Medicine, 101 Daehak-ro, Jongno-gu, Seoul, 03080 Republic of Korea

**Keywords:** Dexmedetomidine, Infant, Congenital heart disease, Cardiopulmonary bypass, Neuroprotection, Randomized controlled trial

## Abstract

**Background:**

Infants undergoing cardiac surgery under cardiopulmonary bypass are vulnerable to postoperative neurodevelopmental delays. Dexmedetomidine has been shown to have protective effects on the heart, kidneys, and brain in animals and adults undergoing cardiac surgery with cardiopulmonary bypass. We hypothesized that dexmedetomidine would have a neuroprotective effect on infants undergoing cardiopulmonary bypass and planned a prospective randomized controlled trial with postoperative neurodevelopment measurements.

**Methods:**

This is a single-center, prospective, double-blinded, randomized controlled trial with 1:1 allocation. A cohort of 160 infants undergoing cardiac surgery with cardiopulmonary bypass will be enrolled. After induction, dexmedetomidine will be infused with a loading dose of 1 μg/kg and a maintenance dose of 0.5 μg/kg/h or the same amount of normal saline will be administered. Upon initiation of cardiopulmonary bypass, an additional dose of dexmedetomidine (0.01 μg/cardiopulmonary priming volume) will be mixed with the cardiopulmonary bypass circuit. The primary outcome will be the proportion of infants who score lower than 85 in any of the cognitive, language, or motor Bayley scales of infant development-III tests 1 year after the surgery. Other feasible outcome measures will include differences in plasma glial fibrillary acidic protein, troponin I, interleukin-6, urinary neutrophil gelatinase-associated lipocalin, and perioperative major adverse events. The results of the Bayley scales of infant development-III test from the study group and the control group will be compared using a chi-squared test under intention-to-treat analysis. A generalized estimating equation will be used to analyze repeated measurements over time.

**Discussion:**

This study will enable us to assess whether the use of dexmedetomidine can alter the early neurodevelopmental outcome in infants undergoing cardiac surgery with cardiopulmonary bypass and also estimate effects of dexmedetomidine on other organs.

**Trial registration:**

ClinicalTrials.gov NCT04484922. Registered on 24 July 2020

**Supplementary Information:**

The online version contains supplementary material available at 10.1186/s13063-022-06217-9.

## Administrative information

Note: the numbers in curly brackets in this protocol refer to SPIRIT checklist item numbers. The order of the items has been modified to group similar items (see http://www.equator-network.org/reporting-guidelines/spirit-2013-statement-defining-standard-protocol-items-for-clinical-trials/).

**Table:** 

Title {1}	The effect of dexmedetomidine on neuroprotection in pediatric cardiac surgery patients: study protocol for a prospective randomized controlled trial
Trial registration {2a and 2b}.	Clinicaltrials.gov (identifier: NCT04484922, date: 24 July 2020)
Protocol version {3}	Version 3.2 (2020.08.24)
Funding {4}	This research was supported by the institutional source (SNU 800-20200331) and the National Research Foundation of Korea funded by the Ministry of Science and ICT, Government of Korea (Grant No.: 2020R1F1A1049067). Expenses for purchasing dexmedetomidine and other consumables, laboratory tests, Bayley scales of infant development were provided.
Author details {5a}	Sang-Hwan Ji, Pyoyoon Kang, In-Sun Song, Young-Eun Jang, Ji-Hyun Lee, Jin-Tae Kim, Hee-Soo Kim and Eun-Hee Kim Department of Anesthesiology and Pain Medicine, Seoul National University Hospital, Seoul National University College of Medicine,101 Daehak-ro, Jongno-gu, Seoul, Republic of Korea
Name and contact information for the trial sponsor {5b}	Seoul National University Hospital +82-2-2072-2623, irb@snuh.org
Role of sponsor {5c}	The trial sponsor is not involved in the study design; collection, management, analysis and interpretation of data; writing of the report; the decision to submit the report for publication, and will not have authority over any of these activities.

## Introduction

### Background and rationale {6a}

Recent advancements in medical procedures have improved the survival rate and life expectancy of children with congenital heart disease (CHD). However, about 50% of patients suffer from cognitive decline, which negatively affects their quality of life and increases medical expenses [1]. Consequently, neurodevelopmental outcomes have been an important issue in the management of CHDs [1–4]. This abnormal neurodevelopment can be innate and due to non-modifiable factors, such as congenital neurologic abnormalities [5], or modifiable factors, including perioperative parameters such as cardiopulmonary bypass (CPB) [6] and anesthesia [7], that mediate the risk of neurodevelopmental impairments after infant cardiac surgery [1, 6]. With regard to modifiable factors, there is an increasing demand for research on neuroprotective strategies, early prediction of neuronal damage via biomarkers, and early intervention [1–3, 5, 8–11].

The ability of anesthetic drugs that act on gamma-aminobutyric acid or N-methyl-d-aspartate receptors to influence neurodevelopment in children has been warned by the Food and Drug Administration [12]. Dexmedetomidine, a selective α_2_ adrenergic receptor agonist that is irrelevant to gamma-aminobutyric acid and N-methyl-d-aspartate receptors, is gaining popularity and has been the subject of many studies [12]. In animal studies, dexmedetomidine is known to reduce anesthesia-induced neurotoxicity [13] and has a protective effect on cerebral ischemic reperfusion injury by reducing neuroinflammation [14–16]. In humans, dexmedetomidine was suggested to inhibit the rise of inflammatory biomarkers during and after CPB in adults [17] and to reduce 1-year mortality and postoperative complications in adults undergoing cardiac surgery [18]. In infants undergoing CPB, although still off-label in most countries, there are reports that dexmedetomidine can be safely administered [19, 20] with the availability of their pharmacokinetic mechanism [19, 21, 22]. However, the benefit of dexmedetomidine on neuroprotection during cardiac surgery in infants is still unclear [23].

### Objectives {7}

We hypothesized that a neuroprotective strategy using dexmedetomidine as an anesthetic aid would reduce events of neurotoxicity due to cardiac surgery in infants. With this hypothesis, we planned a prospective randomized controlled trial to evaluate the effect of dexmedetomidine on neurodevelopmental outcomes in children undergoing congenital heart surgery. The Bayley scales of infant development (BSID)-III is a standardized tool for assessing the development of infants and young children in the cognitive, motor, and language domains [1, 24–26]. It classifies children aged between 0 and 42 months into 17 different age groups and provides the relative score against the average for the age group to which the participant belongs [27]. Children with scores lower than 85, which is one standard deviation below the average, are recommended to undergo early intervention or therapy. We employed the Korean version of the BSID-III test which was validated in several researches and used in Korea for the detection of neurodevelopmental delays [28–32].

We also decided to measure various plasma and urine biomarkers that indicated evidence of organ damage. Glial fibrillary acidic protein (GFAP), a cytoskeleton protein found in astrocytes, is known to be a marker of astroglial cell injury or necrosis. GFAP can be used to diagnose or predict the prognosis of brain injury from trauma or infant cardiac surgery as its plasma level increases [11, 33–37]. Infants are more prone to ischemia-reperfusion injury and tend to show higher troponin I levels compared to older children after CPB [38]. Urinary neutrophil gelatinase-associated lipocalin (NGAL) levels are an early predictive biomarker of acute kidney injury after CPB [39]. Interleukin-6 (IL-6) is an inflammatory cytokine that is an important mediator in the systemic response to CPB. We aim to determine whether dexmedetomidine has the potential to show protective effects against kidney, myocardial, or other organ damage.

### Trial design {8}

This study is designed as a prospective, quadruple-blinded, parallel group, randomized controlled trial with the allocation ratio of 1:1.

## Methods: participants, interventions, and outcomes

### Study setting {9}

This single-center trial is intended to be conducted at the Seoul National University Hospital, a tertiary general hospital located at Seoul, Republic of Korea.

### Eligibility criteria {10}

We plan to enroll infants aged less than a year undergoing open-heart surgery with cardiopulmonary bypass for atrial septal defect, ventricular septal defect, or tetralogy of Fallot without severe hypoxemia. It is known that surgical complexity and cardiopulmonary bypass time affect brain volume and neurodevelopment in infants [40]. Complex CHDs can be categorized from 1 to 5 according to the complexity of the surgical procedure (Society of Thoracic Surgeons-European Association for Cardio-Thoracic Surgery mortality score category) [41]. Consequently, we decided to include patients from within the same category (category 1). Additionally, as cyanosis can affect neurodevelopment, we excluded cyanotic heart disease patients. The detailed exclusion criteria are listed in Table 1.

**Table 1:** Exclusion criteria

History of hypersensitivity to any drug	
Presence of hypotension or bradycardia considering age *Bradycardia (heart rate < 80 beats/min) or hypotension (systolic blood pressure < 70 mmHg for infants, < 60 mmHg for neonates)*	
Elevated liver enzyme levels (AST > 100unit/L or ALT > 50unit/L)	
Need for deep hypothermic circulatory arrest	
Presence of complex cardiac defect	
Single ventricular physiology	
Plan of additional operation within a year	
Preoperative use of beta-antagonists	
Presence or history of any neurological disorder	

*AST* aspartate transaminase, *ALT* alanine aminotransferase

### Who will take informed consent? {26a}

One of the trial members who have medical doctor’s license will explain the study protocol and obtain written informed consent from the parents or guardians of each patient. Participants will be screened and informed consent will be obtained on the day before the surgery.

### Additional consent provisions for collection and use of participant data and biological specimens {26b}

There is no plan for use of participants’ data and biological specimens in ancillary studies.

### Interventions

#### Explanation for the choice of comparators {6b}

Patients will be randomly allocated to the study group and control group. The patients in the control group will receive the same amount of normal saline during the surgery.

#### Intervention description {11a}

##### Study protocol

Patients will be taken to the operating room at the scheduled time of the surgery. Under the monitoring of pulse oximetry for oxygen saturation (SpO_2_), electrocardiogram (ECG), and non-invasive blood pressure, anesthesia will be induced with 0.02 mg/kg of atropine, 5 mg/kg of sodium thiopental, 0.1 mg/kg of midazolam, and 2 μg/kg of fentanyl. Endotracheal intubation will be performed after the administration of 1 mg/kg of rocuronium, followed by arterial and venous catheterization. Patients will be allocated into one of the following two groups: the study group or the control group. For the study group, dexmedetomidine at a concentration of 1 μg/ml will be prepared as the study drug, while a placebo of normal saline will be used in the control group. After arterial and central venous catheterization, infusion of the study drug is initiated after anesthetic induction. For the dosing regimen of dexmedetomidine, we referred to a previous study by Zuppa and colleagues [19] to maintain a plasma concentration of dexmedetomidine between 500 and 700 pg/mL. At the start of the infusion, a loading dose of 1 μg/kg of dexmedetomidine or placebo will be infused for 10 min. Subsequently, a maintenance dose of 0.5 μg/kg/h of dexmedetomidine or placebo will be infused. Upon initiation of cardiopulmonary bypass, an additional dose of dexmedetomidine or placebo (0.01 μg/cardiopulmonary priming volume) will be mixed with the priming volume of the CPB circuit. Infusion of the study drug will be continued until the end of anesthesia.

**Fig. 1:**
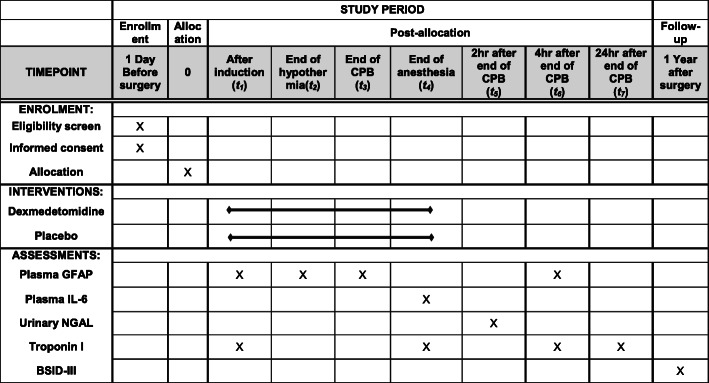
Schedule of patient enrollment, interventions, and assessments

Blood sampling will be performed six times, and a single urine collection will be performed during the study to measure plasma GFAP, IL-6, troponin I, and urine NGAL. Details regarding the schedule of blood and urine collection are shown in Fig. 1.

After surgery, patients will be transferred to the pediatric intensive care unit. Routine postoperative monitoring of ECG, SpO_2_, and invasive blood pressure will be performed, along with laboratory tests, including arterial blood gas analysis, coagulation tests, serum electrolyte levels, blood cell counts, liver function tests, kidney function tests, C-reactive protein levels, and glucose levels. Upon recovery, patients will be transferred to the general ward and discharged. The patients’ parents are then informed about the follow-up visit. Within a month of the day 1 year after the surgery, patients will visit the hospital and undergo the BSID-III test.

##### Anesthetic management

We will monitor the depth of anesthesia using Sedline^®^ (Masimo Corporation, Irvine, CA, USA) and titrate sevoflurane concentration to maintain the patient state index range of 25–50 during the surgery. Regional cerebral oximetry from the left and right forehead will be monitored using O3 sensors (Masimo Corporation, Irvine, CA, USA) and recorded throughout anesthesia. Blood pressure and heart rate will be maintained at ±20% of the preoperatively measured values at the ward. Transfusion of red blood cells, fresh frozen plasma, and platelets will be performed according to our institutional protocol. Packed red blood cells will be transfused when hemoglobin levels are < 8 g/dL in non-cyanotic infants and < 10 g/dL in cyanotic infants. Fresh frozen plasma or platelet concentrates will be administered on the basis of rotational thromboelastometry (ROTEM, TEM International GmbH, Munich, Germany), and a fibrinogen test (FIBTEM) and external test (EXTEM) will be used to obtain data after administration of protamine.

##### CPB management

Non-pulsatile CPB is instituted with high-performance, low-prime volume oxygenators. Crystalloids of plasma solution A, heparin, sodium bicarbonate, antibiotics, and packed red blood cells are included in the priming solution and 5 ml/kg of 20% albumin is added. The total priming volume is 130 mL for neonates and 250–300 mL for infants. The flow rate ranges from 120 to 170 mL/kg/min. The target hematocrit is maintained between 23 and 28%, and pH-stat management is employed during CPB. After weaning from CPB, modified ultrafiltration is performed.

#### Criteria for discontinuing or modifying allocated interventions {11b}

In the case where continuing the study protocol affects the safety of the patient, withdrawal of consent to continue in the study by any of the patients’ parents, or judgment that reliability of the data is compromised, the patient will be dropped from the study. The dropout of an enrolled patient can be decided at any stage of the study. On the occurrence of any unexpected serious adverse event, the study will be suspended until further instructions from the institutional review board. If we fail to enroll more than 30% of the total planned number of subjects, we will consider halt or alteration of the study, including consideration for modifying the inclusion criteria.

#### Strategies to improve adherence to interventions {11c}

Not applicable, the intervention was performed during the general anesthesia.

#### Relevant concomitant care permitted or prohibited during the trial {11d}

Not applicable, there is no relevant concomitant care that is permitted or prohibited.

#### Provisions for post-trial care {30}

The principal investigator has an insurance that is in accordance with the legal requirements in Korea. This insurance provides coverage for any damages to research participants through injury or death caused by the study. The insurance applies to the damages that become apparent during the study or within 1 year after the end of the study.

### Outcomes {12}

Our primary outcome will be the proportion of infants who obtained a score lower than 85 in any of the cognitive, language, or motor domains of the BSID-III test 1 year after the surgery. The raw score for each domain will be compared according to the patient’s age group according to the manual [27], and a converted score will be obtained. Converted scores will be normally distributed with an average of 100 and a standard deviation of 15. Patients’ scores are classified as follows: “average” for score 85 or higher, “at risk” for score 70 to 84, or “delayed” for scores below 70. Secondary outcomes will be categorized as biomarkers measured between induction of anesthesia and 24 h after the surgery, anesthesia profile, and CPB profile during the surgery. The details of the outcome measurements are shown in Table 2.

**Table 2:** Outcome variables

Primary outcome	
Proportion of children with score < 85 in any of the cognitive/ language/motor domains of the BSID-III test	
**Secondary outcomes**	
Biomarkers	
Plasma concentration of GFAP and relationship with BSID results	
IL-6 level after CPB	
Urinary NGAL level after CPB	
Troponin-I level	
Anesthesia profile	
Vital signs	
Depth of anesthesia	
Cerebral oximetry	
Bleeding and transfusion	
Duration of surgery and anesthesia	
Presence of arrhythmia	
CPB profile	
Duration of CPB	
Minimum body temperature during hypothermia	
Duration of rewarming	
Postoperative data	
Duration of mechanical ventilation	
ICU length of stay	
Laboratory data	

*BSID* Bayley Scales of Infant Development, *GFAP* glial fibrillary acidic protein, *IL-6* interleukin-6, *NGAL* neutrophil gelatinase-associated lipocalin, *CPB* cardiopulmonary bypass, *ICU* intensive care unit

### Participant timeline {13}

The schedule of enrolment, interventions, and assessments is depicted in Fig. 1.

### Sample size {14}

In a previous study, 75% of patients who underwent surgery with cardiopulmonary bypass during infancy or cardiac surgery during the neonatal period were marked below 85 at BSID within 3 years [42]. We assumed that dexmedetomidine infusion could reduce the proportion of patients with low BSID from 75 to 50%. With an alpha error of 5% and power of 80%, 132 patients with a 1:1 allocation to the study group and the control group will be required. Considering the relatively long time span between enrollment and follow-up, we set the dropout ratio to be close to 20%. Finally, we plan to enroll 160 patients with a 1:1 allocation ratio to each group.

### Recruitment {15}

Three trial members (S-H. Ji, P. Kang, E-H. Kim) will make list for children who are scheduled to undergo cardiac surgery at the Seoul National University Hospital at least 1 day before the surgery. One of the trial members will visit the candidate’s parent to the ward, check inclusion and exclusion criteria, explain about pros and cons of the study, provide at least 20 min to ponder, and obtain written informed consent from one of the parents.

### Assignment of interventions: allocation

#### Sequence generation {16a}

A sequence for the randomization table was automatically generated from the website (“https://sealedenvelope.com/”). Study patients will be allocated to the study group or control group in a ratio of 1:1.

#### Concealment mechanism {16b}

A nurse who is not participating in the study keeps the randomization table in an isolated cabinet. The allocation is concealed until the last participant’s study protocol has ended.

#### Implementation {16c}

After enrollment of a patient, the nurse who keeps the randomization table identifies the allocation and prepares the study drug accordingly.

### Assignment of interventions: blinding

#### Who will be blinded {17a}

The investigators, care providers, the patient’s guardians, outcome assessors, and data analysts will be blinded to group allocation.

#### Procedure for unblinding if needed {17b}

Unblinding is allowed on occurrence of any serious adverse event that forces the participant to drop out from the study and is determined by the trial steering committee (TSC). When unblinding of a specific patient is decided, the nurse who keeps the randomization table will be asked to review the table and tell the group allocation to the TSC.

### Data collection and management

#### Plans for assessment and collection of outcomes {18a}

As indicators for neurodevelopment, the cognitive, motor, and language domains of the BSID-III test will be assessed [26]. For each domain, the child starts from one of the 17 starting points according to his or her age, which varies from point A to Q in alphabetical order. A detailed table showing the starting points according to age is provided in Additional file 1. If the child fails in any of the first three tasks, the test continues at the starting point which is one level below his or her age. After five consecutive failures, the test ends. For each task, a predefined limit for time or number of trials exists. The patients will be scored according to the age group classification in the BSID-III test. The BSID-III test will be administered by a single, pre-specified examiner for all the participants. The examiner is a nurse from the department of Neonatology who has more than 9 years of testing experience and has a complete understanding of the English and Korean version of the test. Interpretation of the scores are mentioned in the ‘outcomes’ section.

For secondary outcomes, study accruals including results of the BSID-III test; the plasma concentration of GFAP, IL-6, and troponin I; urinary level of NGAL; invasive blood pressure; ECG; SpO_2_; end-tidal concentration of sevoflurane; bispectral index; cerebral oximeter; total anesthesia time; total operation time; total duration of CPB; lowest body temperature; duration of rewarming; duration of mechanical ventilation after surgery; amount of transfusion; estimated blood loss; blood coagulation tests; total intensive care unit length of stay; total hospital stay; and 1-year mortality rate will be collected. Fig. 1 shows the schedule of enrollment, interventions, and assessments throughout the study.

### Plans to promote participant retention and complete follow-up {18b}

To improve adherence to the study protocol, the follow-up measures for the study are planned simultaneously with the standard pediatric and cardiothoracic appointments. Apart from showing up for the follow-up appointments, participants do not need to adhere to specific tasks. For participants, a pre-determined transportation fee will be provided.

### Data management {19}

A web-based data management system is not used. Data for screening will be recorded by trial members P. Kang and S-H Ji. After enrollment, all study data will be recorded to the paper case report form and be entered into a computer for analysis by P. Kang, I-S. Song, Y-E Jang, and E-H Kim. Data will be stored for 3 years after the end of the study. Data accuracy will be checked by the principal investigator of the study.

### Confidentiality {27}

Patients will be referred to only by the participant number. Informed consent forms and paper-based case report forms will be stored separately. Data will be stored within a double-locker until expiration of the storage period and will be only accessible to trial members. After the storage period, the data will be destroyed.

### Plans for collection, laboratory evaluation, and storage of biological specimens for molecular analysis in this trial/future use {33}

Patients’ blood samples for measurement of plasma concentration of GFAP and IL-6 will be immediately stored in an ethylene-diamine-tetra acetic acid (EDTA) tube (BD Vacutainer®, Becton Dickinson Korea, Seoul, Republic of Korea). After centrifuging the samples at 3000 rpm for 10 min, the supernatant will be collected and stored in a sterile internal cryogenic vial (Cryotain^TM^, SCILAB Korea, Seoul, Republic of Korea). The cryovials will be kept in a freezer below −70 °C until analysis. Blood samples for measurement of troponin I and urine samples for measurement of NGAL will also be immediately stored in an EDTA tube and sent to the laboratory in the hospital for analysis. The biological specimen will be discarded after analysis. We will not use the biological specimens in ancillary studies.

### Statistical methods

#### Statistical methods for primary and secondary outcomes {20a}

The proportion from the study group and the control group will be compared using the chi-squared test under per protocol analysis. Statistical methods for secondary outcomes will include independent *t*-tests, Mann–Whitney *U* tests, linear regression, generalized estimating equations, multiple regression models adjusted for potential confounders, chi-squared tests, or Fisher’s exact tests. To evaluate the safety, ECG, SpO_2_, and invasive blood pressure are monitored throughout the study. The appearance of bradycardia (heart rate < 80 beats/min) or hypotension (systolic blood pressure < 70 mmHg for infants and < 60 mmHg for neonates) will be recorded and compared. All statistical analyses will be performed using SPSS® Statistics 23 (IBM, Chicago, IL, USA).

#### Interim analyses {21b}

No interim analysis will be conducted during this trial.

#### Methods for additional analyses (e.g., subgroup analyses) {20b}

Not applicable, no additional analysis intended for this study.

#### Methods in analysis to handle protocol non-adherence and any statistical methods to handle missing data {20c}

Participants who withdraw or terminate from the study will be considered as lost. Reasons for withdrawal or termination will be documented. We expect a withdrawal rate of 20%.

#### Plans to give access to the full protocol, participant-level data, and statistical code {31c}

Sharing the data can be considered by the corresponding author on reasonable request.

### Oversight and monitoring

#### Composition of the coordinating center and trial steering committee {5d}

The coordinating center is composed of the clinicians of the Department of Anesthesiology and Pain Medicine and the Department of Cardiovascular and Thoracic Surgery of Seoul National University Children’s Hospital, including the principal investigator. The TSC consists of the principal investigator and two investigators who are responsible for recruiting the patients and conducting the study. The TSC will check and discuss whether the study is being conducted appropriately the day after enrollment of each patient.

#### Composition of the data monitoring committee, its role, and reporting structure {21a}

A data monitoring committee is not appointed for this study. Instead, safety of the participants, appropriateness of the data, and conduction of the trial will be monitored every 3 months under supervision of the principal investigator and a specific monitoring person.

#### Adverse event reporting and harms {22}

The principal investigator will report any of the serious adverse events to the institutional review board within 15 days of notice. A list of adverse events that are not serious will be reported every 3 months. When reporting adverse events or harms, causality, timing of occurrence, severity, seriousness, provided management will be reported together.

#### Frequency and plans for auditing trial conduct {23}

Auditing the trial will be conducted at any time by the institution’s quality assurance team or the Ministry of Food and Drug Safety of Korea.

#### Plans for communicating important protocol amendments to relevant parties (e.g., trial participants, ethical committees) {25}

Any amendment to the protocol will be reviewed by the institutional review board of the Seoul National University Hospital before application. If there is significant change in the protocol such as inclusion criteria, dosage of the study drug, or frequency of blood sampling, it will be also reported to the Ministry of Food and Drug Safety of Korea for approval. After approval, the changes will also be reflected in the contents of the protocol posted in the registry (https://clinicaltrials.gov). Any deviation from the study protocol will be documented in a separate form and will be reviewed at audits of the trial. Serious deviation from the study protocol will be reported to the institutional review board of the Seoul National University Hospital.

### Dissemination plans {31a}

The results will be disseminated via publication in a peer-reviewed journal, be available at the registry site (https://clinicaltrials.gov/ct2/show/NCT04484922), and presented at scientific meetings.

## Discussion

The purpose of this study is to evaluate the effect of dexmedetomidine in terms of neuroprotection to infants undergoing surgery under cardiopulmonary bypass for congenital heart disease.

Dexmedetomidine, an α-2 receptor agonist, is known to provide a sedative effect with hemodynamic stability, analgesia, and reduced stress response [43]. Major protective mechanisms of dexmedetomidine on organs are by diminishing ischemic injury and reducing systemic inflammatory response [44] and inhibiting apoptotic cell death [45]. High-mobility group box 1 has been nominated to be an early mediator of inflammation and organ damage in ischemia-reperfusion injury by increasing production of tumor necrosis factor-α, IL-1, IL-6, and other pro-inflammatory mediators [46]. Suppression of inflammation by dexmedetomidine after CPB is suggested to be due to inhibition of nuclear factor κB [17]. Although there have been reports that dexmedetomidine may protect the brain during anesthesia or cardiopulmonary bypass [13–16], those studies are limited to rats and not humans. Our study is expected to provide evidence on the neuroprotective effects of dexmedetomidine in infants undergoing cardiopulmonary bypass. The strength of this study is that we will simultaneously measure GFAP, which is a plasma biomarker for brain injury [33, 34, 37], and the BSID-III test which is an actual measurement for neurodevelopment.

Determining an adequate infusion rate of dexmedetomidine is difficult as there is no clear evidence for an appropriate plasma concentration of dexmedetomidine that exerts a neuroprotective effect. Because there are reports that target plasma concentrations of up to 600 pg/mL are safe [47, 48], we will aim to maintain a plasma concentration of 500–700 pg/mL. As Zuppa et al. [19] recently recommended a dosing regimen of dexmedetomidine according to the desired plasma concentration in infants undergoing CPB, we planned a simple and practical regimen in which we can expect the plasma concentration to be around 500–700 pg/mL.

There are reports that BSID-III can underestimate developmental delays [49] and that other methods such as Hammersmith Infant Neurological Examination might be better for infants with CHD [50]. However, we chose BSID-III due to the availability of ample data for reference, including previous reports that examined children with CHD by BSID-III [25]. Moreover, BSID-III was the tool approved in our institution.

Our study has some limitations. First, it would be better if the patients undergo the BSID-III test before the surgery as a baseline. However, we expect our design of a randomized controlled trial would compensate for the lack of baseline data, since the BSID-III was validated as a tool for measuring development of infants and toddlers against their age-appropriate tasks [26]. Also, the Korean version of BSID-III test was validated in several researches and used in national cohort study in Korea to evaluate the neurodevelopment outcome in children [28–32]. Second, measurements of other biomarkers associated with neuroinflammation such as IL-1 or tumor necrosis factor-α are not included in our study.

In summary, we expect this study to provide evidence on the neuroprotective effect of dexmedetomidine in infants undergoing cardiopulmonary bypass and to be a cornerstone for long-term evaluation of those effects.

## Trial status

Protocol version: v3.2 (date: 08/24/2020)

Recruitment of the first patient: 09/16/2020

Number of patients recruited: 33 (date: 10/12/2021)

Expected date for completion of recruitment: 05/31/2023

## Supplementary Information

**Additional file 1.** Start points according to age group in Bayley scales of infant development. Start points of each section according to the participant’s age. If the participant fails in the first three tasks, the test restarts at the point one level below the actual age.

## Abbreviations

CHD: Congenital heart disease
CPB: Cardiopulmonary bypass
BSID-III: Bayley Scales of Infant Development Third Edition
GFAP: Glial fibrillary acidic protein
NGAL: Urinary neutrophil gelatinase-associated lipocalin
IL-6: Interleukin-6
SpO_2_: Oxygen saturation
ECG: Electrocardiogram
TSC: Trial steering committee
EDTA: Ethylene-diamine-tetra acetic acid
