# Supplementary material for: The effect of dexmedetomidine on neuroprotection in pediatric cardiac surgery patients: study protocol for a prospective randomized controlled trial
Source: Trials. 2022 Apr 8;23:271. doi: 10.1186/s13063-022-06217-9 (PMC8991922; doi:10.1186/s13063-022-06217-9)
Supplement: Supplementary file 1 — Additional file 1. Start points according to age group in Bayley scales of infant development. Start points of each section according to the participant’s age. If the participant fails in the first three tasks, the test restarts at the point one level below the actual age. [file 13063_2022_6217_MOESM1_ESM.docx]

**Additional file 1.** Start points according to age group in Bayley scales of infant development. Start points of each section according to the participant’s age. If the participant fail to success first three tasks, the test restarts at the point of one level below. After five consecutive failures, the test ends. The patients will be scored according to this age group classification.

| Age | Start Point |
| --- | --- |
| 16 days – 1 month 15 days | A |
| 1 month 16 days – 2 months 15 days | B |
| 2 months 16 days – 3 months 15 days | C |
| 3 months 16 days – 4 months 15 days | D |
| 4 months 16 days – 5 months 15 days | E |
| 5 months 16 days – 6 months 15 days | F |
| 6 months 16 days – 8 months 30 days | G |
| 9 months 0 days – 10 months 30 days | H |
| 11 months 0 days – 13 months 15 days | I |
| 13 months 16 days – 16 months 15 days | J |
| 16 months 16 days – 19 months 15 days | K |
| 19 months 16 days – 22 months 15 days | L |
| 22 months 16 days – 25 months 15 days | M |
| 25 months 16 days – 28 months 15 days | N |
| 28 months 16 days – 32 months 30 days | O |
| 33 months 0 days – 38 months 30 days | P |
| 39 months 0 days – 42 months 15 days | Q |
